# Traumatic Brain Injury and Sexuality: User Experience Study of an Information Toolkit

**DOI:** 10.2196/14874

**Published:** 2020-03-18

**Authors:** Pascale Marier-Deschênes, Marie-Pierre Gagnon, Julien Déry, Marie-Eve Lamontagne

**Affiliations:** 1 Université Laval Department of Rehabilitation Québec, QC Canada; 2 Centre interdisciplinaire de recherche en réadaptation et intégration sociale Québec, QC Canada; 3 Centre de recherche sur les soins et services de première ligne de l'Université Laval Québec, QC Canada; 4 Université Laval Faculty of Nursing Québec, QC Canada; 5 Centre de recherche du CHU de Québec Québec, QC Canada

**Keywords:** user-centered design, user experience, traumatic brain injury, sexuality, health information

## Abstract

**Background:**

After having sustained a traumatic brain injury (TBI), individuals are at risk of functional impairments in information processing, abstract reasoning, executive functioning, attention, and memory. This affects different aspects of communicative functioning. Specific strategies can be adopted to improve the provision of health information to individuals with TBI, including the development of written materials and nonwritten media.

**Objective:**

A user-centered design was adopted to codevelop four audiovisual presentations, a double-sided information sheet, and a checklist aimed at informing individuals about post-TBI sexuality. The last phase of the project was the assessment of the user experience of the information toolkit, based on the User Experience Honeycomb model.

**Methods:**

Overall, two small group discussions and one individual semistructured interview were conducted with individuals with moderate to severe TBI.

**Results:**

The participants mentioned that the toolkit was easily usable and would have fulfilled a need for information on post-TBI sexuality during or after rehabilitation. They mostly agreed that the minimalist visual content was well-organized, attractive, and relevant. The information was easily located, the tools were accessible in terms of reading and visibility, and the content was also considered credible.

**Conclusions:**

Aspects such as usability, usefulness, desirability, accessibility, credibility, and findability of information were viewed positively by the participants. Further piloting of the toolkit is recommended to explore its effects on the awareness of the potential sexual repercussions of TBI in individuals and partners.

## Introduction

Approximately 69 million individuals globally are victims of a traumatic brain injury (TBI) each year [1]. TBI is the leading worldwide source of morbidity and mortality caused by an injury, and its socio-economic impact is worth billions of dollars annually. After having sustained a TBI, individuals are at risk of functional impairments in information processing, abstract reasoning, executive functioning, attention, and memory, which affect different aspects of communicative functioning [2]. Individuals with severe TBI are especially likely to experience difficulties with understanding and assimilating new information [3,4]. A variety of difficulties related to the ability to read, write, or communicate in general are also common. This means, for example, individuals can struggle with focusing on an individual line of text, or with understanding the content of written and verbal messages. These difficulties can involve low, trauma-related health literacy after hospital discharge, which implies that the individuals do not have the necessary capacity to seek, process, and understand the health information that would help them make informed decisions concerning the medical treatment they receive or their health situation [5,6]. In these cases, health care providers require the ability to shape the information to enhance communication and understanding [6]. To improve the provision of health information to individuals with TBI, the development of written, audio, and visual material is suggested [7,8], as well as the adoption of nonwritten media, like videos [9]. The variety of support solutions to be considered reflects the extent of the interindividual variability in post-TBI consequences that can be experienced.

Many impacts of TBI, including potential damage to the frontal and temporal lobe and its adverse effects on physical, cognitive, behavioral, and emotional functions, can result in sexual difficulties [10,11]. Among the most common repercussions are decreased desire, decreased ability to become excited and maintain excitement, and difficulty or inability to reach orgasm [11,12]. These effects are reported in both men [13-16] and women [15,17] and are more common among individuals with TBI than in the general population [18]. A decrease in the quality of an individual’s sexual life, an increase in their sexual dissatisfaction, and a decrease in their satisfaction with their relationships were also documented [19]. The decrease in the frequency of sexual intercourse [20] is higher in individuals suffering from depression as a result of TBI [21]. For this purpose, depression is considered the most sensitive negative predictor of post-TBI sexual dysfunction [20,22], along with older age of individuals [23]. Conversely, although it is less common, hypersexuality is sometimes observed [24].

However, regardless of its importance for many people with TBI, sexuality remains a rarely discussed topic during rehabilitation [13,25-29]. Studies highlight that most rehabilitation professionals usually take a reactive approach to addressing sexual dysfunction with their patients [30]. The issue is mainly discussed if the individual with TBI or the couple raises concerns. However, informing and educating single patients and couples about post-TBI sexuality is part of a holistic approach to rehabilitation [31]. The typical direct and indirect impacts of TBI on sexual functioning justify the need to inform patients, but very little information is available or had been adapted to this population’s needs. Accordingly, a French information toolkit on post-TBI sexuality was cocreated with individuals with moderate to severe TBI (MSTBI) and their life partners. It includes four audiovisual presentations, each intended for a particular group (single women, women in a relationship, single men, men in a relationship), a double-sided information sheet, and an 18-item checklist of common TBI repercussions on sexuality. The main objective was to develop supporting information material with consideration for post-TBI individuals’ impaired comprehension ability and specific design needs. This involves, for example, repeating the information and adding visual cues to support the retention of information [32]. A detailed description of the cocreation approach, including the choices of form and content for the different tools, is provided elsewhere [33]. Following this process, the user experience was assessed to uncover areas where improvements could be made to the tools. While there is no consensus on the definition of user experience [34], the adopted definition used in this study is the one by the International Organization for Standardization [35]:

A person's perceptions and responses that result from the use and/or anticipated use of a product, system or service.

The objective of this paper is to report on the user experience assessment conducted with individuals with MSTBI. To date, the experience of individuals with MSTBI using information tools is poorly documented. This gap prevents researchers and clinical teams from adequately planning the design of educational materials that could provide accurate and understandable information best suited to this population’s specific needs.

## Methods

### Study Design

A user-centered design [36] was adopted for the larger study to encompass the cocreation process of the information resources [33]. This dynamic and iterative approach involves working with target users throughout the process of developing a product. For the user experience assessment, we carried out two small group discussions and one semistructured interview with individuals who had sustained an MSTBI. We used a semistructured interview guide to explore participants’ experiences of the information toolkit, and we based our interview guide’s questions on Morville’s User Experience Honeycomb model [37]. This simple model presents a honeycomb structure in which seven separate facets of the user experience are identified. It can be used to describe how individuals use, think, and feel about a product. To avoid misinterpretation and to increase the validity of participants with cognitive impairment answers, we designed clear and direct questions based on each subconcept (Textbox 1).

One of the seven facets of the user experience honeycomb model was not documented. The value of the product, which can be assessed by examining if the product advances the mission of the organization behind it, did not apply to our set of tools.

**Questions related to user experience and underlying concepts.**

**Findability**

- Do you easily find the information you are looking for?
- Are the tools easy to navigate?

**Accessibility**

- Is the product designed so that even users with a disability can have the same user experience as others?
- Are reading and viewing tools accessible?

**Usability**

- Are these tools user-friendly and easily searchable?
- Are they simple and easy to use?

**Usefulness**

- Would these tools have filled a need during your rehabilitation?
- Are they useful?

**Credibility**

- Does the information transmitted in the tools seem credible to you?

**Desirability**

- Is the visual aspect of interest to you?
- Is it attractive?

### Participants

A convenience sample of participants was recruited within a regional association of individuals with TBI, following receipt of ethics approval from the Research ethics board of the Centre intégré universitaire de santé et de services sociaux (CIUSSS) de la Capitale-Nationale. A case manager from the association made a preselection of participants based on the following inclusion criteria: have sustained an MSTBI (as documented in the association files), suffered the TBI no more than five years prior, have been discharged from a rehabilitation program, and are considered able, by the case manager, to take part of a discussion in the context of a small group for 45 minutes. An invitation was given to potential participants. Individuals willing to participate were contacted over the phone to confirm their interest and provide additional details.

### Procedure

Small discussion groups were formed based on the participants’ gender and relationship status. Participants were first invited to read the two-sided information sheet on common post-TBI repercussions on sexuality for as long as it took for them to either finish reading or to mention they could not perform the task. They also read the 18 elements of the checklist aimed at supporting individuals in identifying potential issues and raising their concerns with rehabilitation professionals. Then, individuals of the same gender and relationship status watched one of four 14-minute-long audiovisual presentations. Each of these four presentations addressed common issues related to sexuality that can occur after a TBI and was specifically aimed at a particular group (single women, women in a relationship, single men, and men in a relationship). The main subjects covered included the decrease or absence of sexual desire, erectile dysfunction, the decrease or absence of vaginal lubrication, pain, the difficulty or inability to reach orgasm, the inability to fantasize, and the decreased frequency of sexual intercourse. Other aspects that can impact sexuality, such as fatigue, depressive symptoms, lower self-esteem, and sensory deficit, were also discussed. The information sheet offered a summary, and the four narrated presentations provided more extensive information than the sheets. The learning objectives of the toolkit were to increase individuals with TBI’s and their partners’ knowledge of common repercussions of TBI on sexuality, to help them to identify potential solutions to common issues, and to normalize the discussion about sexuality during the rehabilitation process. The participants either watched the presentation as a group or alone. Two computers were at the participants’ disposition. They had control over the computer, and they could playback the parts they did not understand.

Data on the user experience were collected using a purpose-designed interview guide. The first author led both the 45-minute-long small group discussions and the individual interview using a semistructured interview guide. Digital audio recordings were transcribed verbatim by a research assistant.

### Data Analysis

Verbatim transcripts were analyzed using a deductive approach to thematic analysis [38]. The transcripts were first cross-checked with the digital audio recordings and observational notes of the group process. The transcripts were then anonymized and uploaded in Dedoose (SocioCultural Research Consultants, LLC, Los Angeles, California, United States), an online mixed data analysis program. Two members of the research team (PMD and JD) independently read over the transcripts and coded the data according to the six predetermined themes, which included usability, usefulness, desirability, accessibility, credibility, and findability of information. After all the transcripts were coded, the few disagreements in coding were identified. Each was discussed, and a consensus was reached on the appropriate coding. Thus, a high level of intercoder agreement was attained [39]. Then, all quotes that had the same code were uploaded in an excel sheet presenting the participant's number (deidentified information) and the quote itself. The main ideas were identified and synthesized.

## Results

### Overview

Six adults with MSTBI participated in the user experience assessment (Table 1). An individual interview was carried out with one individual who could not be present at the time of the small group discussions.

**Table 1:** Description of the participants.

Participant	Sex	Age	Single	In a relationship
P01	F	42	✓	
P02	M	61		✓
P03	F	40		✓
P04	F	41		✓
P05	F	52		✓
P06	M	42	✓	

### Usability

Usability refers to how well users can learn and use a product to achieve their goals. The participants positively evaluated this aspect, and they mentioned that the toolkit was easily usable. The availability of both the narrated presentations and the printed information supported their comprehension of the content. P04 mentioned that, because of problems related to the treatment and analysis of information, she could not have used the double-sided information sheet alone.

If you give me [this sheet], I will read it, but I won’t understand a thing.

P04

However, she said she understood everything from the narrated presentation, which included much more information. Participants shared their appreciation for the simple use of the web-based Prezi (Prezi Inc, Budapest, Hungary) software.

### Usefulness

Usefulness requires that tools fulfill a direct information need of the target group. Results show that the usefulness of the toolkit was satisfactory. The participants reported that the tools would have met a need for information on post-TBI sexuality during or after rehabilitation. P03 shared that, had she watched the presentation during rehabilitation, she would have known she was not alone when dealing with a specific problem. P06 highlighted that the information presented normalized his personal experience and brought him relief. He had chosen not to ask questions to rehabilitation professionals, being too proud to raise concerns about his condition, but the tools brought him the answers he was looking for. He felt this was a fun way to raise awareness about both post-TBI sexuality problems and their potential solutions. Other participants, like P03, mentioned that partners would benefit from watching the presentation to acknowledge that the described issues are common:

it is important not to be the only ones saying, “I have a problem,” and for our partners to hear this from someone else.

This was perceived positively since it could take the burden of sexual issues off the individuals' shoulders. Even participants who had received information on post-TBI sexuality during rehabilitation thought it provided complementary details. Others said that the toolkit would have supported a discussion with rehabilitation professionals. None of the topics were considered inappropriate or not useful.

### Desirability

Desirability is the quality of a product that evokes emotion and appreciation by its image, identity, brand, or other design elements. The participants mostly agreed that the minimalistic visual content was well organized and attractive. P01 mentioned, though, that the basic aspect of the visual icons, as opposed to detailed pictures, could be modified to be more appealing. However, the same participant added that presenting more sophisticated visual content might distract the audience from the narrated content. She concluded it was preferable to keep it simple. P06 thought the visual aspect was well balanced, being neither too complicated nor too basic

It’s good, it was not childish, and was well presented. It was right on the target.

### Accessibility

Accessibility focuses on how an individual with a disability accesses or benefits from a product. It involves making the information readable and intelligible to the target audience, especially when the public is not a specialist in the field [40]. For individuals with MSTBI, considering their low level of health literacy, this might include the use of an appropriate language level, the availability of an alternative to written material, the presence of significant illustrations, and a consideration of the amount of information presented. The participants thought the information tools were accessible in terms of reading and visuals. The chosen font was easy to read, and the size was considered big enough. They thought the level of language adopted was appropriate and suitable for rehabilitation patients’ consultations. The length of both the presentations and the written information was acceptable to participants. To this regard, P02 mentioned: “I didn’t think it was too long.”

### Credibility

Credibility is the quality of being trustworthy and believable. This means that users need to feel that they can trust the information provided. For this study, the participants considered the information they were provided to be credible. A participant thought that the fact that the content was well explained supported his perception of credibility:

By the way you discuss [the issues], I would trust

the information

### Findability of information

Findability is the ease with which information can be found, but it also involves the capacity to navigate through the toolkit and locate the required information. Well-organized information and the use of color and bold font to designate important words helped participants locate the content relevant to their situation. P06 said he quickly saw the information he was looking for:

When I looked at it earlier, I saw “decreased level of sexual desire” and “difficulty to reach orgasm.” [This made me think] there it is, she is going to talk about what I wanted to talk about. I think it's great.

## Discussion

### Primary Findings

The objective of this study was to assess the user experience of a toolkit on post-TBI sexuality in individuals with MSTBI. The evaluation focused on how well potential users could use the toolkit to meet their information needs on the subject, as well as how satisfied they were with the tools' design. Aspects such as usability, usefulness, desirability, accessibility, credibility, and findability of information were discussed and positively viewed by the participants. These results are encouraging for the dissemination to come. However, before reaching this stage, an evaluation of the acceptability of the tools will be carried out with rehabilitation professionals in the province of Quebec. Afterward, the tools will be made openly accessible through the Clinical practice guideline for the rehabilitation of adults with moderate to severe TBI’s website [41].

To our knowledge, this is the first study to report on the user experience of individuals with MSTBI using post-TBI sexuality information resources. The limited availability of information resources addressing this specific subject might explain the difficulty in locating similar work despite extensive research. In the same vein, a recent scoping review on TBI education for adult patients and families has shown that the available literature on information delivery about TBI and its consequences mostly involves a population with mild TBI [42]. This reinforces our perception that studies similar to our work are still limited, despite the growing recognition of the necessity of an attitudinal shift that would lead to actively supporting both patient and public involvement [43].

The only other information resource on post-TBI sexuality to have been evaluated with documented results in the literature is the booklet entitled “You and Me: A Guide to Sex and Sexuality After Traumatic Brain Injury” [44]. While the user experience of this booklet was not explored, global satisfaction was assessed in a consumer evaluation [45]. A total of 12 individuals with brain injury were interviewed, and they gave a highly positive evaluation of the guide’s practicality, length, informativeness, and the encouraging and comforting information provided. A subsample of eight individuals also had the cognitive ability to provide a dichotomous rating of each chapter of the booklet. These individuals thought all chapters were easy to understand and informative, but they provided a poorer rating of some chapters’ relevance based on their situation or that of individuals in a similar situation (64.1%; 41/64).

Other studies with different populations with cognitive impairments also highlighted that the relevance of the information provided is enhanced by the participation of target users in the development process. Ruel et al conducted a research development project aimed at creating documentation on the services offered in a rehabilitation center for individuals with intellectual disabilities and autism spectrum disorder. Illustrated and plain language service descriptions, developed with and validated by the potential users, led to a better understanding of the services provided. These tools are relevant and useful to professionals for their interventions with users and their families [46]. The importance of using plain language and avoiding jargon was also supported by Sudore and Schinllinger in their study for patients with limited health literacy [47].

In the present study, the fact that the topics covered were based on information needs and expectations expressed by individuals with MSTBI led to a first positive evaluation of the toolkit usefulness. Moreover, the development of audiovisual presentations allowed for individuals with reading difficulty, and information treatment and analysis problems, to benefit from the information. It is hypothesized that these are more likely than conventional, author-driven leaflets and booklets to provide a positive user experience to the end-users.

### Limitations and Future Directions

This study has several limitations. First, a selection bias cannot be ruled out. The selection criterion concerning the participants' ability to take part in a group discussion might have led to the recruitment of individuals who are not representative of the target population. In this regard, the functioning level of the participants may have been higher than that of most adults who sustained a moderate to severe TBI. The lack of representation of ethnic minorities and lesbian, gay, bisexual, transgender, queer (or sometimes questioning), and two-spirited (LGBTQ2+) communities is also worth noting. Our small homogeneous sample, therefore, limits the generalization of these results.

The analysis of qualitative content is, moreover, always open to interpretation, despite the precautions taken to adequately transcribe the interviews, to code their contents with rigor, and to correctly synthesize the main emerging ideas. The absence of prior assumptions and personal interests as to the orientations and aims of the project, however, mitigates the probability of causing confirmation bias.

An additional ongoing step before launching the tools online is the evaluation of the tools’ acceptability by rehabilitation professionals. They will use the tools, when applicable, for six weeks. Participants will then complete an electronic assessment questionnaire consisting of closed-ended questions with Likert scales and open-ended questions, documenting the different subconstructs of acceptability [48,49]. Descriptive statistical analysis will be carried out, and a compilation of the answers to the open questions will be made. This will allow the identification of strengths and areas for improvement from a different point of view. The tools will be modified accordingly, and additional tools could then be created to fulfill specific needs.

### Conclusion

This study reports on a user experience assessment conducted with individuals with MSTBI. Aspects such as usability, usefulness, desirability, accessibility, credibility, and findability of information in a post-TBI sexuality information toolkit were viewed positively by the participants. Further piloting of the toolkit is recommended to explore its effects on the awareness of potential sexual repercussions from TBI in individuals with MSTBI and on their partners. However, this study provides new information on the experience of individuals with MSTBI using information tools. It can be of use to researchers and clinical teams in planning the design of educational material, thus fostering the understanding of rehabilitation-related subjects in this population.

## Abbreviations

CIUSSS: Centre intégré universitaire de santé et de services sociaux
LGBTQ2+: lesbian, gay, bisexual, transgender, queer (or sometimes questioning), and two-spirited
MSTBI: moderate to severe traumatic brain injury
TBI: traumatic brain injury
